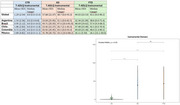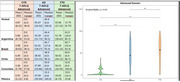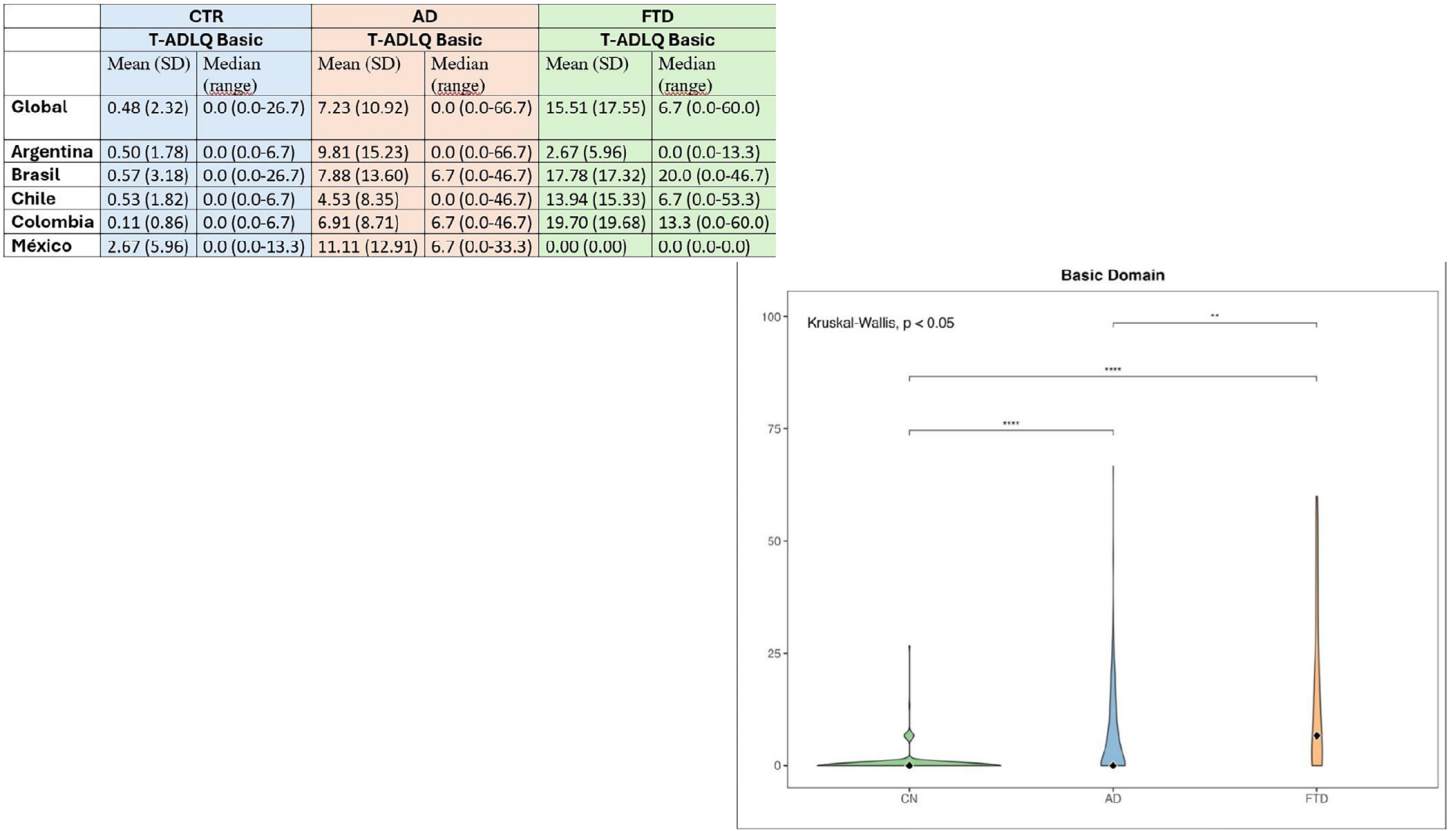# Characterization of functional impairment in Latin American individuals with frontotemporal dementia and Alzheimer's Disease

**DOI:** 10.1002/alz70857_098024

**Published:** 2025-12-24

**Authors:** Patricio Riquelme Contreras, Natalia Santis Alay, Fernando Henriquez, Joaquín Migeot, David Aguillon, Martin Alejandro Bruno, Leonel Tadao Takada, Elisa de Paula, França Resende, Diana L Matallana, José Alberto Ávila Funes, María Isabel Behrens, Nilton Custodio, Bruce L. Miller, Agustin Ibanez, Victor Valcour, Andrea Slachevsky Chonchol

**Affiliations:** ^1^ Geroscience Center for Brain Health and Metabolism (GERO), Santiago, Metropolitana, Chile; ^2^ Neuropsychology and Clinical Neuroscience Laboratory (LANNEC), Physiopathology Department ‐ ICBM, Neuroscience and East Neuroscience Departments, Faculty of Medicine, Universidad de Chile, Santiago, Chile; ^3^ Department of Medical Technology. Faculty of Medicine. Universidad de Chile, Santiago, Chile; ^4^ Memory and Neuropsychiatric Center (CMYN), Neurology Department, Hospital del Salvador and Faculty of Medicine, Universidad de Chile, Santiago, Chile; ^5^ Department of Medical Technology, Faculty of Medicine, Universidad de Chile., Santiago, Metropolitana, Chile; ^6^ Neuropsychology and Clinical Neuroscience Laboratory (LANNEC), Physiopathology Department ‐ ICBM, Neuroscience and East Neuroscience Departments, Faculty of Medicine, Universidad de Chile, Santiago, Metropolitana, Chile; ^7^ Interdisciplinary Center for Neuroscience (NeuroUC) ‐ Laboratory for Cognitive and Evolutionary Neuroscience ‐ Medicine School ‐ Pontificia Universidad Católica de Chile, Santiago, Chile; ^8^ Memory and Neuropsychiatric Center (CMYN), Neurology Service, Hospital del Salvador and Faculty of Medicine, Universidad de Chile, Santiago, Chile; ^9^ Geroscience Center for Brain Health and Metabolism (GERO), Santiago, Chile; ^10^ Latin American Brain Health Institute (BrainLat), Universidad Adolfo Ibañez, Santiago, Chile; ^11^ Center for Social and Cognitive Neuroscience, Universidad Adolfo Ibáñez, Santiago, Chile; ^12^ Neurosciences Group of Antioquia, University of Antioquia, Medellín, Colombia; ^13^ Instituto de Ciencias Biomédicas (ICBM) Facultad de Ciencias Médicas, Universidad Catoóica de Cuyo, San Juan, San Juan, Argentina; ^14^ CONICET, San Juan, Argentina; ^15^ ReDLat, San Juan, Argentina; ^16^ Medical School of University of São Paulo, São Paulo, Brazil; ^17^ Faculdade de Medicina de Ciências Médicas de Minas Gerais, Belo Horizonte, Brazil; ^18^ Global Brain Health Institute (GBHI), University of California San Francisco (UCSF); & Trinity College Dublin, San Francisco, CA, USA; ^19^ Pontificia Universidad Javeriana, Bogota, Cundinamarca, Colombia; ^20^ Instituto Nacional de Ciencias Médicas y Nutrición Salvador Zubirán, Mexico City, DF, Mexico; ^21^ Departamento de Neurología y Neurocirugía, Hospital Clínico, Universidad de Chile., Santiago, Región Metropolitana de Santiago, Chile; ^22^ Facultad de Medicina, Universidad de Chile, Santiago, Región Metropolitana de Santiago, Chile; ^23^ Centro de Investigación Clínica Avanzada (CICA), Universidad de Chile, Santiago, Región Metropolitana de Santiago, Chile; ^24^ Unit Cognitive Impairment and Dementia Prevention, Peruvian Institute of Neurosciences, Lima, Peru, Lima, Lima, Peru; ^25^ Global Brain Health Institute, University of California, San Francisco, San Francisco, CA, USA; ^26^ Memory and Aging Center, Weill Institute for Neurosciences, University of California, San Francisco, San Francisco, CA, USA; ^27^ Global Brain Health Institute (GBHI), University of California San Francisco (UCSF); & Trinity College Dublin, Dublin, Ireland; ^28^ Memory and Aging Center, University of California San Francisco, San Francisco, CA, USA; ^29^ Neuropsychology and Clinical Neuroscience Laboratory (LANNEC), Physiopathology Department – Institute of Biomedical Sciences (ICBM), Neuroscience and East Neuroscience Departments, Faculty of Medicine, Universidad de Chile, Santiago, Chile, Santiago, Chile; ^30^ Neurology Service, Department of Medicine, Clínica Alemana‐Universidad del Desarrollo, Santiago, Chile., Santiago, Chile

## Abstract

**Background:**

Functional impairment (FI) in activities of daily living (ADL) is a critical criterion for diagnosing dementia that could be categorized into its three dimensions: basic (BADL), instrumental (IADL) and advanced activities (aADL). While FI has been well studied in Alzheimer's disease (AD), this characterization has been underexplored in frontotemporal dementia (FTD) and even more in Latin American countries, where genetic and exposome diversity defines variability in its clinical presentation.

**Method:**

We evaluated 547 individuals from RedLat consortium for dementia research in Latin America (262 controls, 236 AD and 49 FTD) from 5 Latin American countries (Argentina, Brazil, Chile, Colombia and Mexico). We performed descriptive statistics for sociodemographic variables. Functional performance was evaluated through the Tecnology‐Activities of Daily Living (T‐ADLQ) global score and subscores for BADL, IADL, and aADL. Kruskal‐Wallis and Dunn post‐hoc tests were performed to compare T‐ADLQ scores between the three groups.

**Result:**

AD participants showed poor global cognitive performance compared to FTD, and FTD presented higher scores in NPI than AD. In total, basic, instrumental, and advanced T‐ADLQ scores, FTD showed higher scores than AD participants, which means poor functional performance in FTD participants; however, these differences only showed significance in the basic domain.

**Conclusion:**

In Latin American individuals, FTD showed poorer functional performance than AD patients, statistically significant in the basic domain. Further research would be oriented toward exploring the relationship between this functional performance and cognitive and clinical symptoms to determine the functional profile in FTD, compared with AD.